# Patterning amyloid-β aggregation under the effect of acetylcholinesterase using a biological nanopore - an *in vitro* study

**DOI:** 10.1016/j.snr.2023.100170

**Published:** 2023-07-08

**Authors:** Nandhini Subramanian, Brittany Watson, Chen-Zhong Li, Melissa Moss, Chang Liu

**Affiliations:** a Biomedical Engineering Program, University of South Carolina, Columbia, SC 29208, USA; b Biomedical Engineering Program, School of Medicine, The Chinese University of Hong Kong, Shenzhen 518172, China; c Department of Chemical Engineering, University of South Carolina, Columbia, SC 29208, USA

**Keywords:** Nanopore, Aβ aggregation, Acetylcholinesterase

## Abstract

Aggregation of amyloid-β peptide (Aβ) is hypothesized to be the primary cause of Alzheimer’s disease (AD) progression. Aβ aggregation has been widely studied using conventional sensing tools like emission fluorescence, electron microscopy, mass spectroscopy, and circular dichroism. However, none of these techniques can provide cost-efficient, highly sensitive quantification of Aβ aggregation kinetics at the molecular level. Among the influences on Aβ aggregation of interest to disease progression is the acceleration of Aβ aggregation by acetylcholinesterase (AChE), which is present in the brain and inflicts the fast progression of disease due to its direct interaction with Aβ. In this work, we demonstrate the ability of a biological nanopore to map and quantify AChE accelerated aggregation of Aβ monomers to mixed oligomers and small soluble aggregates with single-molecule precision. This method will allow future work on testing direct and indirect effects of therapeutic drugs on AChE accelerated Aβ aggregation as well as disease prognosis.

## 1. Introduction

Alzheimer’s disease (AD) is one of the most common neurodegenerative diseases, imposing an enormous challenge in diagnosis and treatment, as the actual cause of the pathogenesis is uncertain. The amyloid hypothesis has been widely accepted as a basis for the development of AD therapeutics [1]. In AD, the amyloid-β (Aβ) peptide forms aggregates that deposit as plaques around neuronal cells and brain vasculature. This deposition is associated with the degradation of neuronal function, leading to impaired memory and cognition, such as compromised thinking, speaking, writing, and other day-to-day activities [2]. Ample *in vitro* experiments have been reported to scrutinize and understand Aβ aggregation [3–6]. Many studies have revealed several natural proteins in the brain that promote the aggregation of Aβ, accelerating the pathology progression in the AD brain. Such proteins include apolipoprotein E (apoE) [7], α_1_-anti-chymotrypsin [8], heparan sulfate proteoglycans [9], and acetylcholinesterase (AChE) [10,11].

**Fig. 1.**
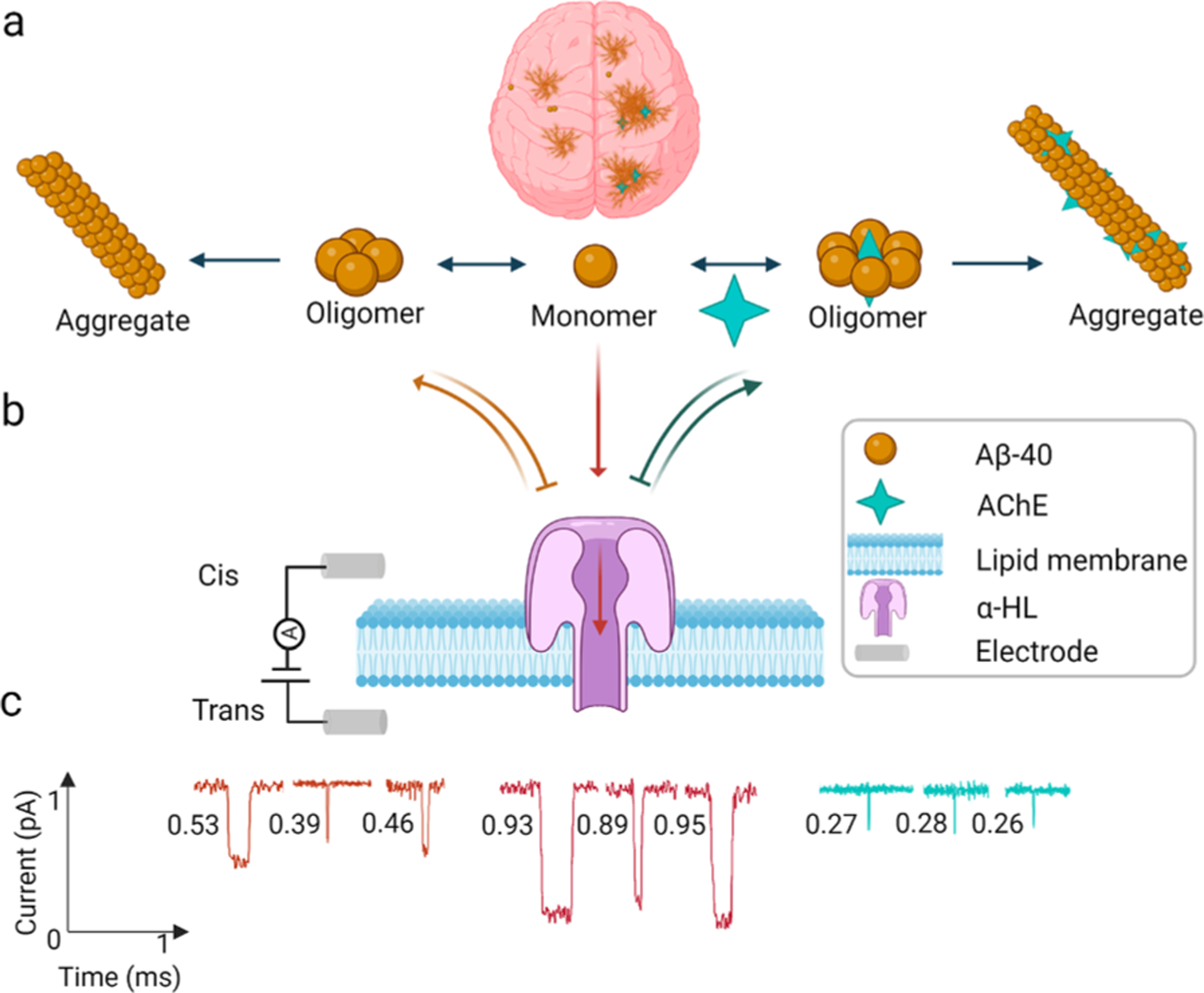
a: Schematic representation of an AD brain showing disease pathology of plaque deposition (left) and expedited plaque deposition due to the AChE enzyme (right), juxtaposed with Aβ–40 and AChE accelerated Aβ–40 aggregation kinetics at the molecular level. b: Interactions of various Aβ–40 and AChE embedded Aβ–40 species with the α-HL nanopore. c: Characteristic signals of oligomer (orange) with current blockade in the range of 0.3–0.7 and dwell time ≥0.3 ms; monomer (red) with current blockade ≥0.7 and dwell time ≥0.3 ms; and small soluble aggregate (cyan) with current blockade ≤0.3 and dwell time in the range of 0.2–0.3 ms.

Among these, the cholinergic enzyme, AChE, becomes an interesting research candidate to address the disease, as it can directly affect the aggregation of Aβ. The primary function of the AChE enzyme is to degrade the neurotransmitter acetylcholine in the synaptic cleft [12]. However, AChE promotes Aβ aggregation due to the interaction between the hydrophobic, peripheral anionic binding site (PAS) on AChE and the cationic sequence of Aβ peptides. This interaction results in the formation of oligomers and insoluble large Aβ aggregates by forming an embedded complex structure (Fig. 1a). In addition, loss of AChE activity has been observed in the diseased cases due to this association between AChE and Aβ [13–16]. Insoluble Aβ fibrils complexed with AChE impose high neurotoxicity in the brain [11]. This implies the importance of a detailed understanding of the interaction between AChE and Aβ, in order to develop effective strategies for inhibiting disease progression.

In previous studies, conventional techniques such as emission fluorescence, circular dichroism, and electron microscopy were used to show the effect of AChE on Aβ aggregation [17–21]. However, none of these techniques provides sensitive and efficient quantitative characterization on the molecular-level. On the other hand, resistive pulse nanopore sensing technology presents tremendous advantages in identifying, analyzing, and characterizing various DNA and protein sequences at the single-molecule level [22–26]. Taking advantage of the high sensitivity of biological nanopore sensors, we envision quantitatively tracing, with single-molecule precision, the accelerating effect of AChE on Aβ aggregation dynamics. In general, an applied electric potential difference drives individual target analyte molecules to translocate through the sensing region of the nanopore, which is embedded inside a stable lipid bilayer separating the electrolytic solution into two chambers. Based on the current blockade depth (I/I_0_), caused by the occlusion of a translocating molecule in the sensing region, and the dwell time, indicating its duration inside the nanopore, the size, charge, and other properties of the analyte can be characterized [24–27]. Herein, we use the widely employed α-hemolysin (α-HL) pore-forming protein to sense and characterize Aβ peptides. As the constriction of an α-HL nanopore is ~1.4 nm in diameter, when 40 residue Aβ (Aβ–40) monomers, oligomers, and small aggregates approach the *cis* head of the nanopore (Fig. 1b), monomers with a diameter less than ~1.4 nm will translocate through the pore, giving distinct translocating signals (Fig. 1c (red)), whereas mixed oligomers of different sizes will exhibit partial translocating signals and partial bumping signals (Fig. 1c (orange)). Owing to their bigger size, significantly more bumping signals will be observed for small aggregates (Fig. 1c (cyan)). This offers us the advantage of quantifying the distributed population at the molecular level, which is challenging using conventional biosensing methodologies like ELISA, gel electrophoresis, or spectroscopic technology. Following our earlier effort in mapping the aggregation kinetics of Aβ–40 over time [28], here, we examine and pattern the kinetics of monomeric Aβ–40 aggregation into different oligomers and small aggregates, both in the absence and presence of AChE at various time points, using robust α-HL nanopores. We observe accelerated aggregation dynamics for Aβ–40 incubated with the AChE promoter enzyme, relative to that of Aβ–40 alone, affirming the potential application of sensitive nanopore biosensors to trace and pattern Aβ aggregation effectively and precisely.

## 2. Materials and methods

### 2.1. Materials

Aβ–40 was purchased from Peptide 2.0 (Chantilly, VA, USA). Recombinant human AChE (expressed in HEK 293 cells, lyophilized powder, ≥1000 units/mg protein by Lowry), α-hemolysin (α-HL) from *Staphylococcus aureus* (lyophilized powder, protein ~60% by Lowry, >10,000 units/mg protein), potassium chloride (KCl), and Tris–HCl were purchased from Sigma-Aldrich. Buffer solutions in all experiments were prepared using deionized water (DI) from a Milli-Q water purification system (with 18.2 MΩ/cm resistivity, 25 °C, Millipore Corporation) and filtered through a 0.02 μm filter before every use.

### 2.2. Preparation of buffer

The work solution of 1 M KCl and 10 mM Tris–HCl was prepared by dissolving KCl (7.46 g) and Tris–HCl (121 mg) in 80 mL of DI water. The pH of the buffer was adjusted to 8.0 by using NaOH (2 M) and HCl (2 M), and made up to 100 mL with DI water to obtain the final electrolyte of 1 M KCl buffered in 10 mM Tris–HCl (pH 8.0).

### 2.3. Purification of Aβ–40

Lyophilized crude Aβ–40 was stored at 20 °C until reconstitution, at which time the peptide was dissolved in 50 mM NaOH. Aβ–40 was purified, and preexisting aggregates were removed by size exclusion chromatography (SEC) on a Superdex 75 HR10/300 column (GE Healthcare, Piscataway, NJ), equilibrated in 100 mM HEPES (pH 7.4) and pretreated with 2 mg mL^−1^ bovine serum albumin to reduce nonspecific interactions between the peptide and the column matrix. The isolated monomer was eluted in 100 mM HEPES (pH 7.4). Concentrations of isolated monomers were determined via UV absorbance (277 nm) with a calculated extinction coefficient of 1450 L mol^−1^ cm^−2^. Aβ–40 monomer was diluted to a concentration of 50 μM and immediately used for experiments.

### 2.4. Preparation of aggregation solutions

To examine the effect of AChE on Aβ–40 aggregation kinetics, 50 μM purified Aβ–40 monomer (in HEPES, pH 7.4) was incubated in the presence or absence of AChE enzyme in a molar concentration ratio of 200:1[21] at 37 °C for two weeks to allow aggregation. An aliquot was removed from the incubation at every fixed time point and tested using the biological nanopore sensor.

### 2.5. Nanopore fabrication and electrical resistive pulse sensing

All single-channel recordings were performed on a Planar Lipid Bilayer (PLB) workstation (Warner Instruments) at room temperature (~23 °C). We followed a previously reported α-HL nanopore fabrication method [29–31]. In brief, an orifice (200 μm in diameter) punctured on a 25 μm thick Delrin wall that separates the *cis* (grounded) and the *trans* chambers of the flow cell was deliberately precoated with 1:10 hexadecane/pentane to support the lipid bilayer membrane. After securing the Delrin cup inside the two-welled flow cell, both chambers were filled with 1 mL of work solution. The Ag/AgCl electrodes were positioned in the chamber, where the bias source electrode was placed on the *trans* side of the cup, and the reference electrode was placed in the grounded *cis* chamber. To form a lipid bilayer membrane, 20 μL (10 mg/mL) of 1,2 diphytanoyl-sn-glycero-3-phosphocholine (Avanti Polar Lipids), dissolved in pentane, was added to the *cis* chamber to allow self-assembly in the orifice. Following this, 100 mV electrical potential was applied to the *trans* chamber using embedded Ag/AgCl electrodes. The membrane capacitance was maintained between 120 and 200 pF following the Montal-Mueller method. Transmembrane potentials were applied from the *trans* chamber via Ag/AgCl electrodes and were held by the potentiostat at 100 mV throughout the experiment.

To attain a stable α-HL nanopore channel, we directly added an aliquot of α-HL protein (~0.05 μg) (Sigma-Aldrich) to the work solution in the *cis* well. When 100 mV *trans* voltage was applied, a single nanopore can self-assemble onto the lipid bilayer and exhibit an open pore current between 90 and 100 pA. To examine the stability and consistency of the pore, both positive and negative voltages (±100 mV) were applied, and the corresponding channel current was recorded. A stable α-HL pore shows a larger current output under a positive *trans* voltage than a negative voltage [22]. After confirming a robust α-HL nanopore insertion from the observed open pore current value, 50 μL of the aggregating analyte solution (Aβ–40 or Aβ–40 with AChE) was added to the *cis* chamber for the subsequent measurement.

### 2.6. Data reading and analysis

After adding analytes to the *cis* chamber, the ionic current recording was performed using a patch clamp amplifier on the PLB workstation with an attached high-pass Bessel filter (cutoff: 5 kHz) at a holding potential of 100 mV. Following the sample addition, the work solution was gently pipetted to facilitate the homogenous distribution of the sample in the *cis* chamber prior to signal recording. For each time point, 3 experimental replicates were performed using a fresh α-HL nanopore for each replicate to study the aggregation kinetics. Digidata 1440A analog-to-digital converter was used to sample the ionic current at 100 kHz, which was processed on the Clampfit 11 software (Molecular Devices). The raw nanopore sensorgram data were analyzed using an inhouse Matlab based algorithm to discern the current blockade and the dwell time for each translocation event. These are the two discriminating properties that characterize various species translocating through the nanopore sensing region. The current blockade represents the capture and translocation of a single molecule through the nanopore constriction, given by I/I_0_ (I = I_0_ I_b_; I_b_: the average current measured in the presence of analytes inside the pore; I_0_: the average baseline current in the absence of analytes). Dwell time indicates the effective interaction time an analyte spent inside the nanopore while translocating through the narrow sensing region before leaving the vestibule. A cut-off current of about 20 pA from baseline and 0.2 ms dwell time was used to identify and eliminate events raised by electrical noise. Results processed by the Matlab algorithm were confirmed by manual inspection.

### 2.7. Statistical analysis

To trace the aggregation kinetics of each analyte (Aβ–40 and Aβ–40 + AChE), I/I_0_ (current blockade) was plotted against dwell time for each time point analysis using Python. The Python modules used for the 2D scatter plots and histograms were Matplotlib and Originlab. To distinguish Aβ–40 monomers from oligomer and small aggregate species, 3 repeated nanopore experiments at fixed intervals were conducted and manually inspected. We then categorized each species by current blockade and dwell time values of signals. Signals with ≥0.7 current blockade and ≥0.3 ms dwell time values characterize monomer species; signals with 0.3–0.7 current blockade and ≥0.3 ms dwell time values characterize mixed oligomer species; signals with ≤0.3 current blockade and dwell time values in the range of 0.2–0.3 ms were attributed to small soluble aggregate species. Relative monomer percentage was calculated as the number of observed monomer signals divided by the total number of observed events: percentage of monomer = (number of monomer events / number of all events) × 100%. The percentage of oligomers and small aggregates were calculated using a similar manner and plotted using Origin 2018. Histograms of the current blockade and dwell time versus the relative event frequency for all experiments were also generated in Origin 2018. To evaluate the effect of AChE’s interference with the signals of the target analyte, box and whisker plots for the current blockade and dwell time of the blank solution (no analyte) and the AChE stock solution containing AChE enzyme alone (0.25 μM) were constructed.

## 3. Results and discussion

### 3.1. Investigating AChE interference on monomer, oligomer, and small aggregate signals

**Fig. 2.**
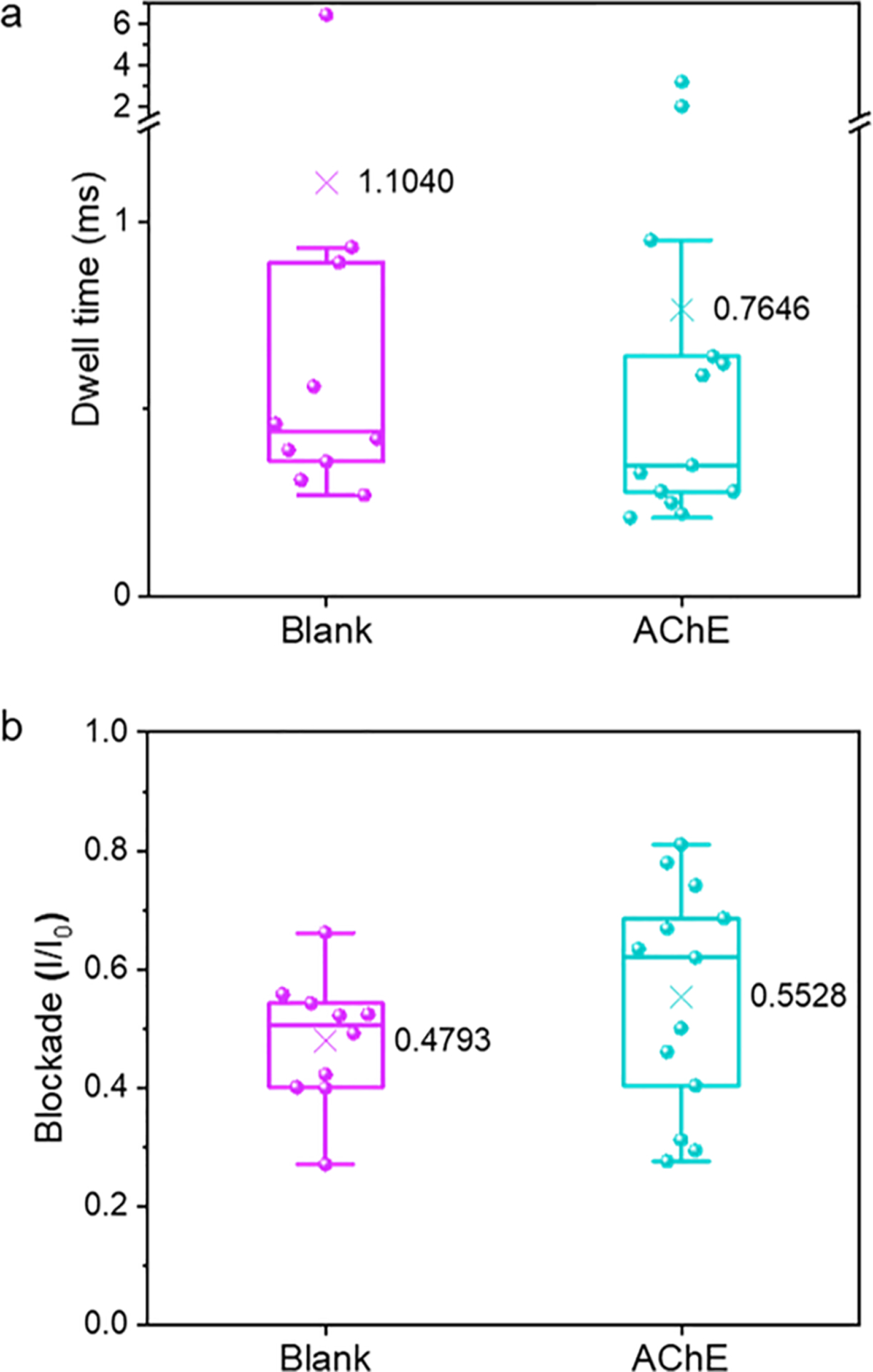
Comparison of dwell time (a) and current blockade (I/I_0_) (b) between blank (no analyte) and AChE (enzyme alone) control experiments observed for 300 s. Each dot represents the dwell time and the blockade value of an observed nanopore event. The ‘X’ mark indicates the mean of the respective data distribution.

Because of its large molecular size, AChE is not expected to translocate the α-HL pore but may generate bumping signals when it interacts with the pore entrance. These non-translocating bumping signals may interfere with the desired oligomeric and small aggregate Aβ–40 bumping signals, potentially compromising the selectivity of our nanopore sensor. Hence, we carried out the interference study of AChE by analyzing two control experiments: (1) the blank work solution with no analyte and (2) the work solution containing AChE alone using the same α-HL nanopore. We first recorded the blank sample measurement for 300 s. Following this, we deliberately added AChE without affecting the stability of the lipid membrane and the pore and continued the electrical recording for another 300 s. Eligible translocation events observed in these two experiments were compare and analyze for differences between dwell time distribution (Fig. 2a) and current blockade distribution (Fig. 2b). We recorded 10 and 13 total signal events for blank and AChE analyses, respectively. Among the observed total events, 4 signals correspond to non-translocating events under each condition. The dwell time distribution of blank and AChE control analyses showed a mean of 1.1040 ms and 0.7646 ms, respectively, and the two-sample *t*-test with *p* = 0.57 (>0.05) indicated no significant difference between blank and AChE. The calculated means of current blockade for blank and AChE experiments are 0.4793 and 0.5528, respectively, and the two-sample *t*-test with *p* = 0.29 (>0.05) showed no significant difference between the two groups. These observations show a negligible difference between the two control experiments indicating insignificant interference of AChE to our subsequent experiments to understand AChE accelerated Aβ aggregation.

### 3.2. Mapping aggregation dynamics of Aβ–40 peptides

To characterize the AChE accelerated Aβ–40 aggregation kinetics, we first recorded the aggregation dynamics of the Aβ–40 peptide over ascending time points as the reference experiment [28, 32, 33]. We incubated monomeric Aβ–40 at a physiological temperature (37 °C) over two weeks and tested with nanopore sensors. Aβ exhibits concentration- and time-dependent aggregation dynamics [34]. Very slow aggregation of Aβ–40 was reported at physiological concentrations (pM to nM) [35–39]. To efficiently achieve sufficient measurable aggregation for examining the nanopore sensors’ ability to quantify and pattern the aggregation dynamics, we used a higher concentration (50 μM) of Aβ– 40. Monomer, mixed oligomer, and small aggregate distributions were characterized based on the current blockade (I/I_0_) and dwell time of the signals. To quantify the monomer population in the reference sample, we tested an aliquot of Aβ–40 solution after 1 h of incubation using α-HL. To trace the ascending aggregation dynamics of Aβ 40, we tested an aliquot of the sample at 1 h, 24 h, 48 h, 72 h, 120 h, 168 h, 192 h, and 288 h of incubation, respectively.

**Fig. 3.**
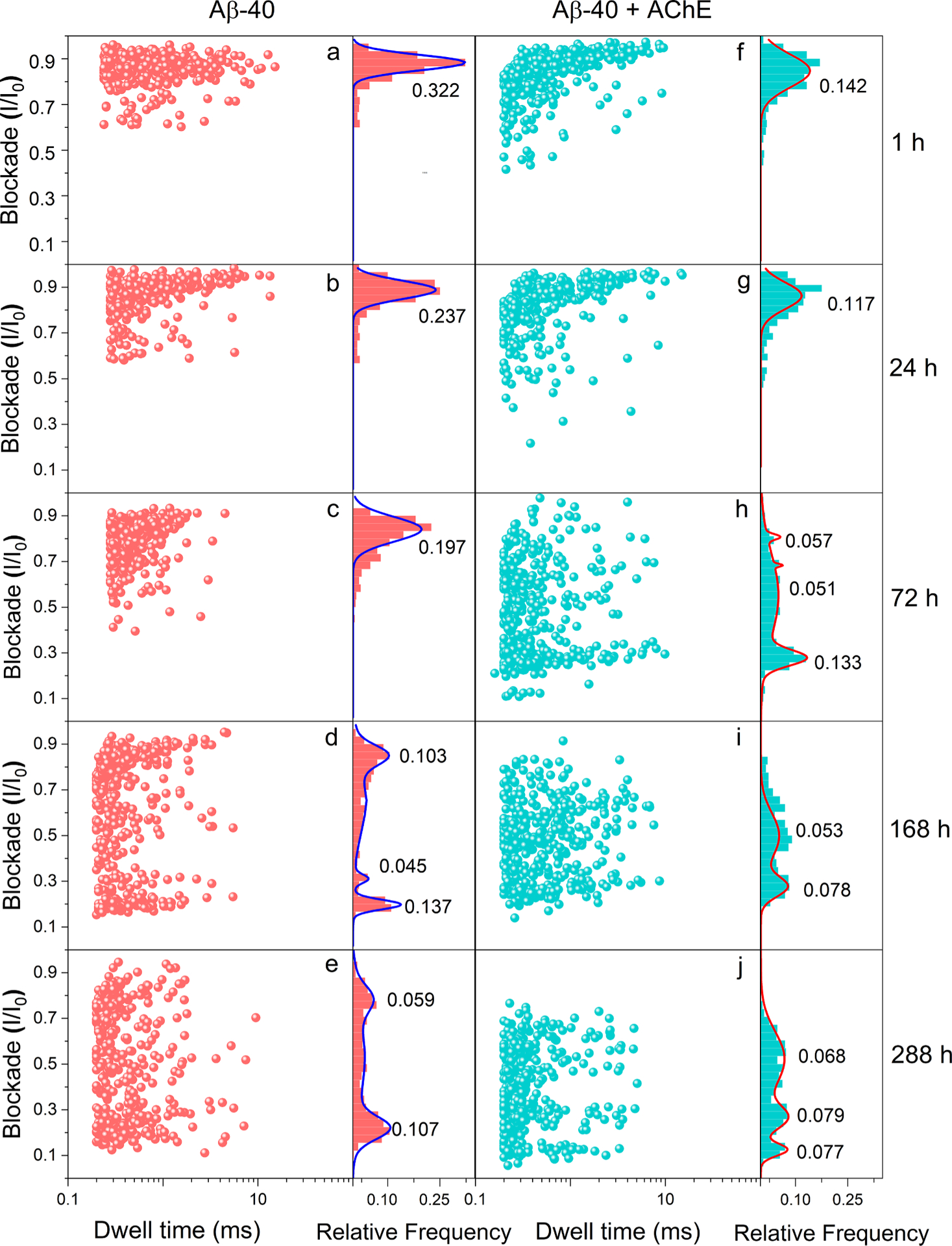
a–e: 2D scatter plots of current blockade versus dwell time of valid events (*N* = 400) from the nanopore recordings of Aβ–40 aggregation at 1 h (a), 24 h (b), 72 h (c), 168 h (d), and 288 h (e). Right: Histograms of I/I_0_ for the respective time point analyses with Gaussian fitting. f–j: 2D scatter plots of current blockade versus dwell time of valid events (*N* = 400) from the nanopore recordings of AChE accelerated Aβ–40 aggregation at 1 h (f), 24 h (g), 72 h (h), 168 h (i), and 288 h (j). Right: Histograms of I/I_0_ for the respective time point analyses with Gaussian fitting. The parameter indicated in the Gaussian fitting is peak height.

2D-scatter plots of current blockade vs. dwell time were made from 400 valid signals from each nanopore recording (Fig. 3 (a–e)). We observed abundant full translocating events corresponding to monomers in the 1 h sample characterized by the deep current blockades and long dwell time. In addition, we also observed some bumping signals (shallow current blockade and short dwell time) due to the presence of low amount of oligomers formed by aggregation (Fig. 3a) [15]. After 24 h, when tested with the nanopore, we noticed comparably fewer translocating events and more bumping events caused by the increasing oligomer concentration. In accordance with the observation, a 2D scatter plot for 24 h analysis showed fewer deep current blockades and an increasing number of small dwell time signals (Fig. 3b). To further elucidate the distinction in the characteristic signals of various molecular populations, we established histograms of relative event frequency against the current blockade with Gaussian fitting (Fig. S1) and dwell time with exponential fitting (Fig. S2) for 400 events at each time point. Similarly, the increasing trend of mixed oligomer and small aggregate events and decreasing monomeric signals were observed from the nanopore measurements at ascending time points. These trends were further characterized by the change of the frequency of dominant events from monomer to mixed oligomer to small aggregate, visualized by the peak shifting in histograms of current blockade upon increasing incubation time from 1 h to 288 h (Fig. S1). Histograms of relative event frequency against dwell time also showed decreasing large dwell time events caused by the depletion of monomers and increasing small dwell time signals due to newly formed oligomers and small aggregates (Fig. S2). After completing the reference studies (Aβ–40), we then incubated Aβ–40 with promoter enzyme AChE to examine the influence on aggregation.

### 3.3. Patterning AChE accelerated Aβ–40 aggregation dynamics

Even a trace amount of AChE was reported to potentially affect Aβ aggregation, increasing neurotoxic fibril deposition and memory deterioration [15,40,41]. At physiological concentrations of Aβ–40, AChE with activity levels in the milli units/mL range can accelerate the aggregation of Aβ–40 [42]. However, the time scale of the aggregation process is still too large for *in vitro* studies. To achieve AChE accelerated Aβ–40 aggregation in a timely manner to demonstrate our nanopore sensor, we incubated 50 μM Aβ–40 with AChE at an optimized 200:1 molar ratio [21] and allowed samples to aggregate over two weeks at 37 °C. During the incubation, an aliquot from the aggregating sample was pipetted out and tested with the nanopore at different time points (1 h, 24 h, 48 h, 72 h, 120 h, 168 h, 192 h, and 288 h). Within 1 h, we observed a significant abundance of translocating signals with long blockade depth and dwell time corresponding to monomers and fewer bumping oligomer signals (shallow blockade and short dwell time). Following this, ascending time point analyses of the incubated solution showed a trend of increasing bumping signals and decreasing translocation signals, similar to the Aβ–40 alone experiment. By analyzing eligible nanopore signals (total number of valid events, *N* = 400) at all time points, histograms were established for relative event frequency against current blockade with Gaussian fitting (Fig. S3) and dwell time with exponential fitting (Fig. S4) to characterize and trace the aggregation profile of AChE accelerated Aβ–40 aggregation. A downfield shift of I/I_0_ was observed in the fitted Gaussian peak center in the relative event frequency, representing the aggregation of free monomers into oligomers and small aggregates over time. An analogous change was observed for dwell time as the relative frequency of long dwell time events decrease with the aggregation over time. To demonstrate the AChE accelerated aggregation of Aβ–40, we presented 2D scatter plots of the blockade against dwell time for selected time points (1 h, 24 h, 72 h, 168 h, and 288 h) of Aβ–40 analyses (Fig. 3(a–e)) and Aβ–40 with AChE analyses (Fig. 3 (f–j)) juxtaposed with corresponding current blockade versus relative frequency histograms on the right side of each panel. The vertical perusal of the nanopore results obtained for Aβ–40 and Aβ–40 + AChE at a fixed aggregation period shows the descending blockade value profile with ascending aggregation hours. In the Aβ–40 condition, we recorded a high frequency of higher blockade at 1 h with a peak height of 0.322, which subsequently reduced to 0.197 at 72 h with a gradual widening of the frequency distribution and down-trending peak center. Later at 168 h and 288 h, we observed a substantial widening of the frequency distribution exhibiting the presence of monomers, mixed oligomers, and small aggregates in the sample. A reduced small aggregate frequency height was observed at 288 h compared to 168 h. This oscillation may be due to the off-pathway oligomerization. These unstable off-pathway intermediates constantly reorder themselves by incorporating or releasing small monomers [43–48]..

**Fig. 4.**
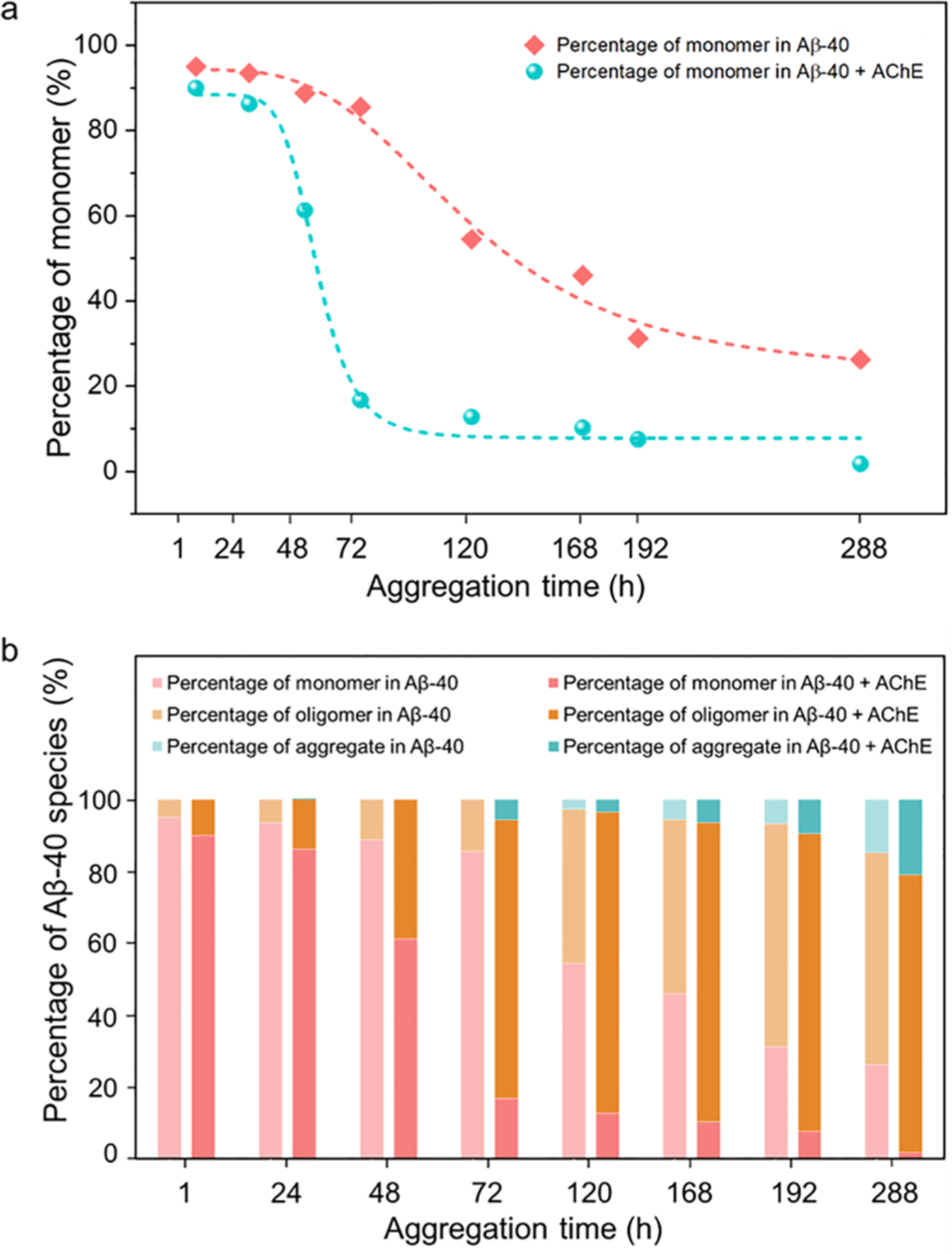
a: 2D scatter plots of percentage of monomer vs. aggregation time to pattern the aggregation kinetics in both Aβ–40 and Aβ–40 + AChE analyses. Trendlines indicate logistic fittings and were used to calculate the power constant, ‘p’. b: Histograms of the percentage of different Aβ–40 species (monomer (red), oligomer (orange), and small aggregates (cyan)) in Aβ–40 (first column) and Aβ–40 + AChE (second column) analyses for the respective time points. Monomer, oligomer, and small aggregates were differentiated based on the blockade and dwell time values, as described in the text.

For Aβ–40 alone aggregation, the observed monomer translocating and oligomer bumping event frequencies for each time point range from 60 to 300 events/minute. For Aβ–40 + AChE analysis, a significantly reduced frequency range of 5–100 translocating and bumping events per minute was observed. This is likely due to the presence of AChE that promotes the aggregation of peptides, affecting both their size and hydrophobicity and resulting in altered interactions with the nanopore. With the ascending incubating time of Aβ–40 and Aβ–40 + AChE analyses, detection frequencies diminished significantly due to decreasing monomers and increasing aggregate sizes. Such observations also evidently exhibit the AChE accelerated Aβ–40 peptide aggregation. To realize the detection of each single molecule of each Aβ–40 species, in the nanopore recordings at each time point, a small aggregate event is defined by the current blockade value of less than 0.3, with the dwell time ranging between 0.2 and 0.3 ms. In signals with a dwell time above 0.3 ms, a blockade value over 0.7 pA defines a monomer, and a blockade in the range of 0.3–0.7 denotes an oligomer. As described in the statistical analysis section, the percentage of monomers, oligomers, and aggregates were calculated based on these specific ranges. A decreasing percentage of monomers was observed at each time point of Aβ–40 and Aβ–40 + AChE (Fig. 4a). Because of the AChE promoting effect, a decreased monomer frequency was observed in Aβ–40 + AChE analyses at 48 h, whereas in Aβ–40 analyses, a significant monomer decrease was observed only after 72 h. The trendlines for the monomer depletion in Aβ–40 and Aβ–40 + AChE analyses indicate power constants (p) of 3.24 and 6.58, respectively. A higher p value indicates a faster decrement rate of the analytes, which is also visualized by a steeper slope of the logistic trendline. The higher p value observed in Aβ–40 + AChE analyses indicates the AChE accelerated Aβ–40 aggregation kinetics. Fig. 4b shows the fragment of each Aβ–40 species with or without the AChE effect at each time point of aggregation. Aβ–40 + AChE analyses show small aggregates within 72 h, while Aβ–40 analyses exhibited small aggregates at 120 h aggregation. In addition, at 288 h, Aβ–40 + AChE showed ~26% greater quantity of oligomers compared to Aβ–40 alone. These results demonstrate that the α-HL nanopore can effectively sense and characterize the aggregation dynamics of AChE accelerated Aβ aggregation.

## 4. Conclusion

The amyloid hypothesis is the leading theory in AD pathogenesis. Naturally available AChE can directly promote the aggregation of Aβ. We demonstrated the accelerated aggregation kinetics of Aβ–40 in the presence of AChE using a robust α-HL nanopore biosensor. Nanopore biosensors offer a simple, sturdy, highly sensitive, and cost-efficient sensing tool to analyze protein conformational changes with single-molecule precision. With this foundation, in the future, we will investigate the effect of potential therapeutic compounds like enzyme-inhibiting drugs [49–51] and aggregation inhibitors on the aggregation kinetics of Aβ. These investigations may aid in better design and evaluation of therapies for AD. Yet it is challenging to distinguish among different-sized oligomers of Aβ with biological nanopores. We are currently developing different-sized solid-state pores capable of identifying different-sized oligomers to enable more sophisticated monitoring of Aβ aggregation and the effect of prospective therapeutics.

## Supplementary Material

Supplementary Information

## Data Availability

Data will be made available on request.
