# Supplementary material for: Patterning amyloid-β aggregation under the effect of acetylcholinesterase using a biological nanopore - an in vitro study
Source: Sens Actuators Rep. Author manuscript; Available in PMC 2023 Dec 1. (PMC10469531; doi:10.1016/j.snr.2023.100170)
Supplement: Supplementary Information [file NIHMS1926645-supplement-Supplementary_Information.docx]

**Patterning Amyloid-β Aggregation under the Effect of Acetylcholinesterase using a Biological Nanopore - an *in vitro* Study**

Nandhini Subramanian ^a^, Brittany Watson ^a^, Chenzhong Li ^b^, Melissa Moss ^a,c^, Chang Liu ^a,c,*^

^a^ *Biomedical Engineering Program, University of South Carolina, Columbia, SC 29208, USA*

^b^ *Biomedical Engineering Program, School of Medicine,* *The Chinese University of Hong Kong, Shenzhen, 518172, China*

^c^ *Department of Chemical Engineering, University of South Carolina, Columbia, SC 29208, USA*

*Address correspondence to: E-mail: [changliu@cec.sc.edu](mailto:changliu@cec.sc.edu)





**Figure S1.** Histogram of relative event frequency versus current blockade (I/I_0_) of all valid events (N = 400) from nanopore measurements of all time points of the Aβ-40 aggregation analysis at 37ºC. The fitting lines were established using Gaussian fitting with parameters indicating peak height.





**Figure S2**. Histogram of relative event frequency versus dwell time of all valid events (N = 400) from nanopore measurements of all time points of the Aβ-40 aggregation analysis at 37ºC. The fitting lines were established using exponential fitting with parameters indicating time constant.





**Figure S3**. Histogram of relative event frequency versus current blockade (I/I_0_) of all valid events (N = 400) from nanopore measurements of all time points of the Aβ-40 + AChE aggregation analysis at 37ºC. The fitting lines were established using Gaussian fitting with parameters indicating peak height.





**Figure S4**. Histogram of relative event frequency versus dwell time of all valid events (N= 400) from nanopore measurements of all time points of the Aβ-40 + AChE aggregation analysis at 37ºC. The fitting lines were established using exponential fitting with parameters indicating time constant.
